# Utilizing Circadian Heart Rate Variability Features and Machine Learning for Estimating Left Ventricular Ejection Fraction Levels in Hypertensive Patients: A Composite Multiscale Entropy Analysis

**DOI:** 10.3390/bios15070442

**Published:** 2025-07-10

**Authors:** Nanxiang Zhang, Qi Pan, Shuo Yang, Leen Huang, Jianan Yin, Hai Lin, Xiang Huang, Chonglong Ding, Xinyan Zou, Yongjun Zheng, Jinxin Zhang

**Affiliations:** 1 Department of Medical Statistics, School of Public Health, Sun Yat-sen University, Guangzhou 510080, China; zhangnx7@mail2.sysu.edu.cn (N.Z.); panq33@mail2.sysu.edu.cn (Q.P.); yangsh223@mail2.sysu.edu.cn (S.Y.); huanglen@mail3.sysu.edu.cn (L.H.); yinjn@mail2.sysu.edu.cn (J.Y.); dingchlong@mail2.sysu.edu.cn (C.D.); zouxy9@mail2.sysu.edu.cn (X.Z.); 2 Zhongshan Center for Disease Control and Prevention, Zhongshan 528400, China; zslinhai@126.com; 3 Sanxiang Community Health Service Center of Zhongshan, Zhongshan 528463, China; mynameishx@163.com

**Keywords:** heart rate variability, electrocardiography, machine learning, composite multiscale entropy

## Abstract

Background: Early identification of left ventricular ejection fraction (LVEF) levels during the progression of hypertension is essential to prevent cardiac deterioration. However, achieving a non-invasive, cost-effective, and definitive assessment is challenging. It has prompted us to develop a comprehensive machine learning framework for the automatic quantitative estimation of LVEF levels from electrocardiography (ECG) signals. Methods: We enrolled 200 hypertensive patients from Zhongshan City, Guangdong Province, China, from 1 November 2022 to 1 January 2025. Participants underwent 24 h Holter monitoring and echocardiography for LVEF estimation. We developed a comprehensive machine learning framework that initiated with preprocessed ECG signal in one-hour intervals to extract CMSE-based heart rate variability (HRV) features, then utilized machine learning models such as linear regression (LR), Support Vector Machines (SVMs), and random forests (RFs) with recursive feature elimination for optimal LVEF estimation. Results: The LR model, notably during early night interval (20:00–21:00), achieved a RMSE of 4.61% and a MAE of 3.74%, highlighting its superiority. Compared with other similar studies, key CMSE parameters (Scales 1, 5, Slope 1–5, and Area 1–5) can effectively enhance regression models’ estimation performance. Conclusion: Our findings suggest that CMSE-derived circadian HRV features from Holter ECG could serve as a non-invasive, cost-effective, and interpretable solution for LVEF assessment in community settings. From a machine learning interpretable perspective, the proposed method emphasized CMSE’s clinical potential in capturing autonomic dynamics and cardiac function fluctuations.

## 1. Introduction

Cardiovascular disease (CVD) remains one of the leading causes of disability and death worldwide [1]. Hypertension, a major risk factor for CVD, is on the rise worldwide [2]. Long-term hypertension can lead to changes in structure and function of heart [3]. These issues can reduce left ventricular ejection fraction (LVEF) and increase risk of arrhythmias and heart failure [4]. LVEF is crucial for doctors to assess cardiovascular health [5,6]. But traditional LVEF measurement methods, such as echocardiography and MRI, require not only complex equipment but also specialized staff [7]. It limits their use in resource-limited community settings. Hence, there is a strong urge for a non-invasive, affordable, and convenient method to estimate LVEF.

Electrocardiogram (ECG) is one of the most commonly used cardiac tests in clinics. It is non-invasive, convenient, and provides real-time recordings, reflecting cardiac activity [8]. Heart rate variability (HRV) analysis, based on ECG signals, quantifies variation in RR intervals (time between consecutive R peaks). It can uncover subtle changes in cardiac autonomic nerve function [9]. Furthermore, HRV analysis from Holter monitoring can enable long-term continuous monitoring of cardiac function in patients, making it suitable for large-scale community screening [10].

Among HRV analysis methods, multiscale entropy (MSE) stands out as an advanced nonlinear dynamic analysis tool [11]. Traditional HRV analysis methods, such as time-domain and frequency-domain analyses, focus mainly on single-scale features and may miss complex multiscale information in ECG signals [12]. MSE can comprehensively consider variation in HRV signal at different timescales. This can more fully reflect complexity and dynamics of HRV, and thus more sensitively capture abnormal changes in cardiac autonomic nerve function [13]. Composite multiscale entropy (CMSE) further enhances estimation accuracy of MSE and addresses its uncertainty issues [14]. However, the application of CMSE in assessing cardiac function in hypertensive patients remains unexplored.

Inspired by aforementioned research, this study aims to explore the use of 24 h Holter monitoring data to systematically develop a predictive model based on CMSE features and a machine learning framework. The goal is to establish an automated method for quantitatively assessing LVEF in hypertensive patients. The study introduces an hourly HRV analysis method, aiming to identify the optimal ECG-based detection time window by analyzing circadian HRV patterns and their dynamic relationship with cardiac function. It is anticipated that this research will address methodological gaps in CMSE applications for hypertensive cardiac function assessment, while providing a reliable, non-invasive, and clinically interpretable alternative for cardiac function evaluation in community healthcare scenarios.

## 2. Method

### 2.1. Dataset

The study cohort (Dynamic ECG & Blood Pressure for Hypertension at Sanxiang, DEBPHAS) consisted of managed care hypertension cases within the primary care community of Zhongshan City, Guangdong Province, China, from 1 November 2022 to 1 January 2025. All patients voluntarily consented to participate after being informed about the study’s procedures and aims. Written informed consent was obtained from each participant. The study was approved by the Public Health School of Sun Yat-sen University (Approval No. 081/2021) and was carried out in compliance with the Ethical Standards of the Declaration of Helsinki.

Participants diagnosed with hypertension were invited to participate. A total of 200 patients met the inclusion and exclusion criteria and were included in the study. The inclusion criteria were as follows: (1) age 18 or above, (2) with a documented history of hypertension from county-level or higher-level hospitals, (3) possessing at least basic communication skills. The exclusion criteria were the following: (1) permanent pacemaker implantation, (2) dextrocardia.

Eligible patients maintained routine activities while undergoing 24 h 12-lead Holter monitoring (CardioCare H1201 Holter recorder; Nalong Technology, Xiamen, China) at a sampling rate of 250 Hz. The protocol included a 10 min supine rest in a quiet environment before ambulatory recording, followed by echocardiography within seven days (refer to previous study settings [15]). All procedures were performed by experienced cardiovascular and ultrasound specialists, with LVEF measured via Simpson’s biplane method (ASE/EACVI guidelines [16]). Baseline characteristics of hypertensive patients are listed in Table 1.

### 2.2. Data Processing

**Figure 1:**
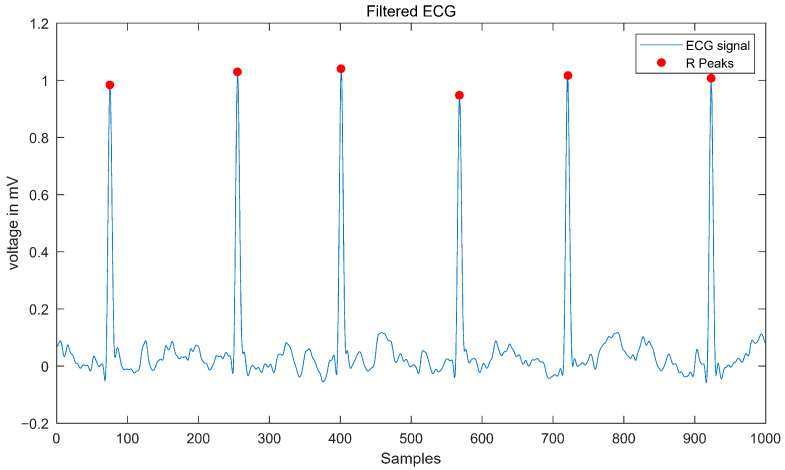
QRS wave detection.

In this study, ECG records of each subject were selected for further processing and analysis. Signal processing involved filtering and wave detection. In this study, we applied a median filter, a 120 Hz low-pass filter, a 50 Hz notch filter, and a 0.3 Hz high-pass filter. We also implemented a phase-free stationary wavelet transform (SWT) using a Haar wavelet for further noise reduction [17,18]. R-peaks were identified, and multi-lead delineation—specifically, QRS cancelation—was executed for each lead using an open-source MATLAB toolbox [19] (see Figure 1).

### 2.3. HRV Features

The features were extracted using MATLAB R2023a software. A total of 15 basic HRV features were derived from the corrected RR series. The selected HRV features encompassed nonlinear domain characteristics. We utilized several entropy-based methods from the family of entropy measures, including power spectral entropy (PsdEn) [20], energy entropy (Een) [21], approximate entropy (ApEn) [22], sample entropy (SampEn) [23], fuzzy entropy (FuzzyEn) [24], permutation entropy (PEn) [25], attention entropy (Aen) [26], bubble entropy (BubbEn) [27], dispersion entropy (DispEn) [28], distribution entropy (DistEn) [29], gridded distribution entropy (GdEn) [30], incremental entropy (IncrEn) [31], phase entropy (PhaseEn) [32], slope entropy (SlopEn) [33], and symbolic dynamic entropy (SyDyEn) [34], as detailed in Table 2. Generally, these entropy estimators quantify the unpredictability of the RR intervals; higher entropy values indicate greater unpredictability in the RR series. All extracted basic HRV features were then analyzed at multiple scales.

CMSE analysis is a method used to estimate physiological signals across various timescales and to assess the predictability of time series [14]. It involves two main steps: (1) coarse-graining signals into different timescales, and (2) quantifying the degree of irregularity in each coarse-grained time series using entropy measures, as described previously. To construct a coarse-grained series for time series analysis, the data are initially divided into *N*/*τ* non-overlapping windows, each of length *τ*. The data points within each window are then averaged. For a given time series {1 ≤ *j* ≤ *N*}, the coarse-grained series is calculated as follows:

$$y_{k,j}^{\left(\tau \right)}=\frac{1}{\tau }\sum_{\left(j-1\right)\tau +k}^{j\tau +k-1}x_{i}$$

Here, *τ* is the scale factor, 1  ≤ * j*  ≤  *N*/*τ*, and *k* represents the *k*-th coarse-grained time series for a scale factor of *τ*, where 1 ≤ *k* ≤  *τ*. Additionally, the complex patterns of a time series are divided into long-term and short-term curves. In this study, different scales were beneficial for clinical categorization, with CMSE curve up to a scale factor of 20 were significantly useful. The scale factors were further categorized into five different parameters (The calculation formulas are in Supplementary Materials):

1. The value of entropy at scale 1.
2. The value of entropy at scale 5.
3. Slope 1–5: The linear-fitted slope between scales 1 and 5.
4. The area under the curve between scales 1 and 5 (Area 1–5), which serves as a measure of complexity across short timescales, also known as the short-term Complexity Index (CI).
5. The area under the curve between scales 6 and 20 (Area 6–20), which serves as a measure of complexity across long timescales, also known as the long-term CI.

### 2.4. Machine Learning Framework

In this study, we evaluated ten regression models using the previously described 78 features, which comprised 75 HRV features (5 CMSE parameters × 15 basic HRV features), age, sex, and hypertension history. The models assessed included Gaussian Process Regression (GPR) [35], K-Nearest Neighbors (KNNs) [36], linear regression (LR) [37], Multilayer Perceptron (MLP) [38], random forest (RF) [39], Support Vector Machine (SVM) with three kernel functions—linear, Polynomial, and Radial Basis Function (RBF) [40], Treebag [41], and Convolutional Neural Networks (CNNs). The regression model was structured as a CNN with two convolutional layers, each followed by batch normalization (BN) and a rectified linear unit (ReLU). A max pooling layer was used after ReLU to reduce feature dimensions and accelerate training. The consecutive convolutional layers had a kernel size of (2, 1) and 16 filters. The Adam optimizer was chosen with a learning rate of 0.001, L2 regularization of 0.0001, and a decay rate of 0.90. A Leave-One-Out Cross-Validation (LOOCV) approach was implemented to avoid overlap between training and testing data. Recursive Feature Elimination (RFE) was employed for feature selection to identify the optimal subset of up to 25 features (based on 12.5% of the study population of 200). In this method, the step size was set to 1, adding one feature per iteration. In each iteration, features were added one by one to the previous combination, and the set with the lowest RMSE was retained. The process stopped when adding more features failed to reduce RMSE, yielding the most effective feature combination. Model performance was evaluated by root mean square error (RMSE) and Mean Absolute Error (MAE). LVEF estimates were validated using the Bland–Altman method. The median duration of Holter monitoring for patients was 23 h (interquartile range: 23–24 h). Missing HRV feature values were imputed using multiple imputation. The R packages “caret”, “BlandAltmanLeh”, and “mice” were used for machine learning, RFE, Bland–Altman analysis, and multiple imputation, respectively.

### 2.5. Explainability and Visualization

To further evaluate model performance and interpretability, we analyzed feature importance to quantify the contribution of each feature in reducing model error, such as RMSE. The varImp function in the “caret” package was used to calculate feature importance, with methods tailored to the specific model type and input parameters. For instance, in linear regression models, standardized regression coefficients were used, where features with larger absolute coefficients were considered more important for model predictions [42]. Furthermore, SHAP value was also employed for variable interpretability evaluation.

To provide a comprehensive assessment of the model’s predictive ability, we visualized the estimation process using circular heatmaps and lollipop charts. These visualizations were designed to assess and illustrate the model’s performance from multiple perspectives. All analyses and visualizations were conducted using R software (version 4.4.1), with the R packages “caret”, “circlize”, “fastshap”, and “ComplexHeatmap” employed for feature importance analysis, SHAP analysis, and circular heatmaps, respectively.

## 3. Results

### 3.1. Recursive Feature Elimination

**Figure 2:**
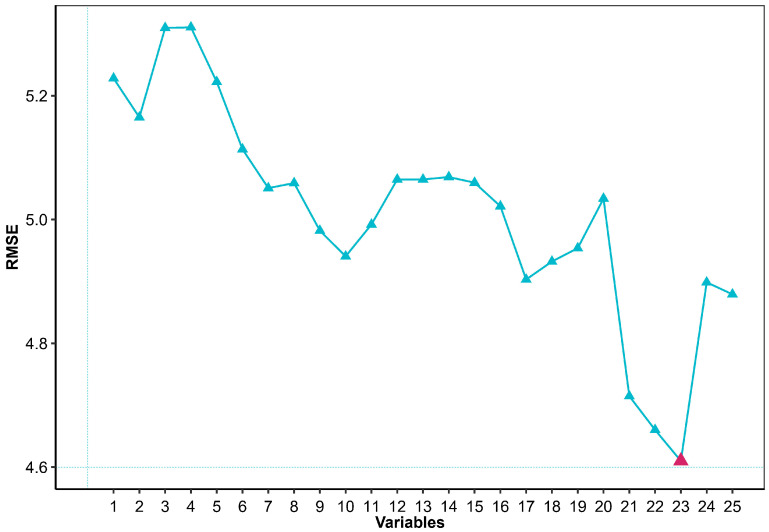
RMSE corresponding to feature sets for 20:00–21:00 time interval utilizing LR model. Red triangle marks iteration at which feature addition ceases.

The optimal feature set selection process is depicted in Figure 2 for the hourly periods from 20:00 to 21:00, utilizing the LR model. During this interval, RMSE reduced as features were gradually incorporated into the set, reaching a minimum at iteration 23 (RMSE = 4.61%), indicating that 23 features were incorporated into the optimal feature set for this hour.

### 3.2. Model Performance

Table 3 presents the average RMSE for LVEF estimation across 24 h periods, using various regression models with their most effective feature subsets. This table offers a comparative overview of how model performance varies throughout the day. It enables an assessment of each model’s suitability for LVEF estimation at different time intervals. Each row corresponds to a specific one-hour interval within 24 h cycle, while each column represents a distinct ML model. The RMSE reflects the average magnitude of prediction errors between estimated and actual LVEF levels for each interval and model. Lower RMSE corresponds to smaller errors and higher prediction accuracy. Similar trends were observed for MAE, as summarized in Table 4.

**Figure 3:**
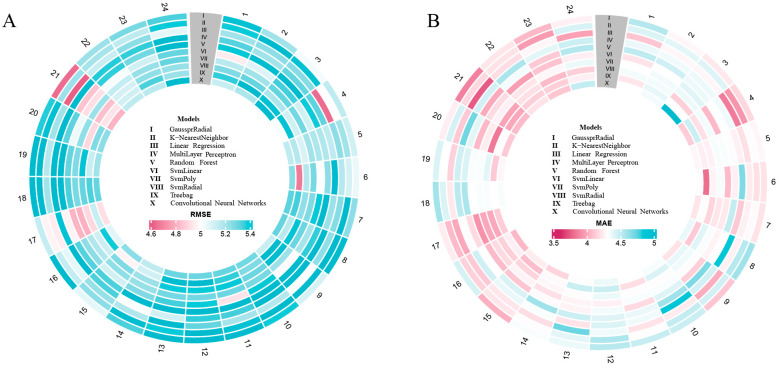
Model performance fluctuates throughout the 24 h period and differs across various regression models with the best feature subset. The heatmaps highlight the best-performing regression models for each hour of the day. Time points are arranged in ascending order, progressing clockwise. Model performance is visualized based on RMSE (**A**) and MAE (**B**) (red is better, blue is worse). The numbered circles (I–X) correspond to the ten regression models evaluated in the study.

The lowest error (RMSE = 4.61%) was achieved between 20:00 and 21:00 using the LR model. The Support Vector Machine with Polynomial Kernel (SvmPoly) model performed best during the afternoon period (16:00–17:00), with a RMSE of 4.80%. The late night (03:00–04:00) and early morning hours (05:00–06:00) showed the lowest errors using the LR and Treebag models, respectively. Additionally, Figure 3 illustrates the distribution of RMSE (A) and MAE (B) across the 24 h period for the ten regression models. Time intervals with the lowest RMSE and MAE values are highlighted in red, indicating that the LR model outperformed other models during two specific intervals.

### 3.3. Bland–Altman Analysis

**Figure 4:**
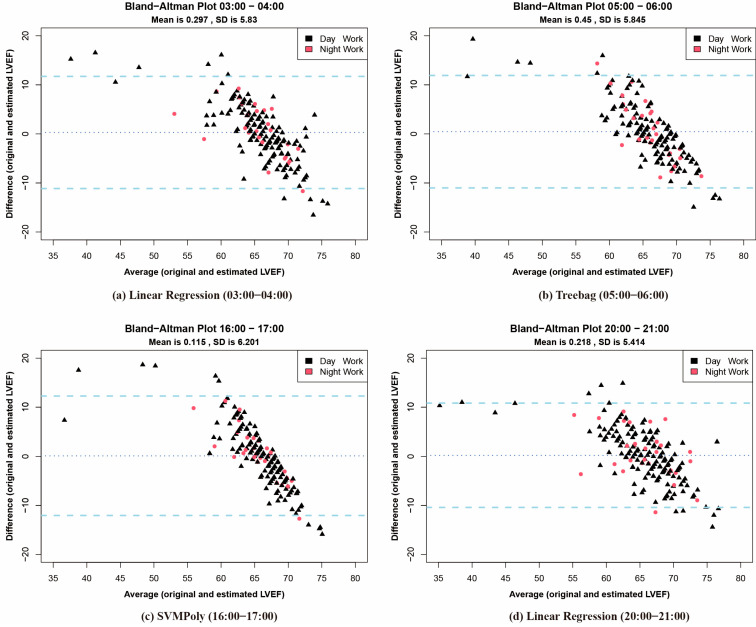
Bland–Altman plots for four specific hours using LR (**a**), Treebag (**b**), SVMPoly (**c**), and LR (**d**), respectively. Differences were computed as predicted minus actual LVEF values. The light blue dotted lines represent the 95% confidence intervals. The legend indicates different types of work (red for night work, black for day work).

To further evaluate the prediction model’s performance, Bland–Altman plots (Figure 4) compare original and estimated LVEF level during the four time intervals with the lowest RMSE: 3:00–4:00, 5:00–6:00, 16:00–17:00, and 20:00–21:00. Notably, patients with night work exhibited a narrower dispersion range, with most LVEF prediction values falling within the 95% limits of agreement. This indicates that automated prediction techniques achieved higher accuracy for individuals engaged in night work during these intervals.

### 3.4. Feature Importance

**Figure 5:**
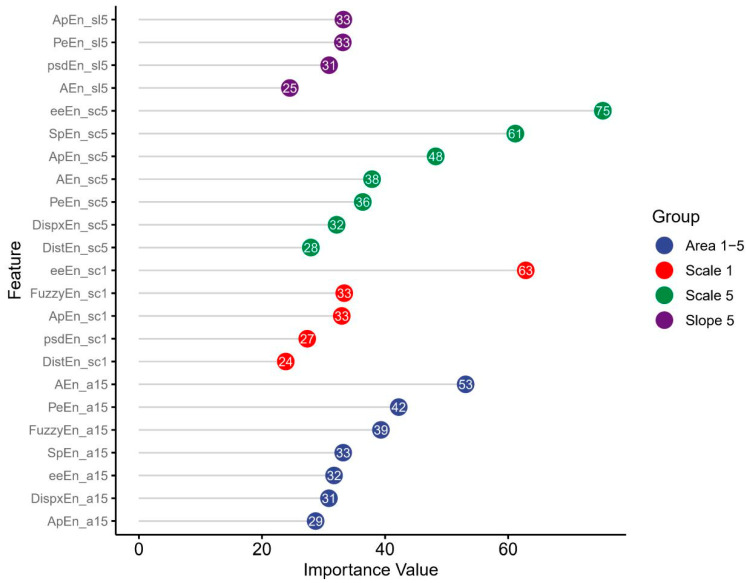
Feature importance value corresponding to the best feature sets for the 20:00–21:00 time interval based on the LR model. To better visualize the importance of different CMSE-based HRV feature categories to the model, the lollipop chart is organized in two stages: first, sorted by the total scores of individual features, and then grouped and sorted within each category. Features are divided into four distinct color-coded groups: Scale 1, Scale 5, Slope 1–5, and Area 1–5. The y-axis lists the features derived from various CMSE parameters, arranged within each category from the most to least important. The x-axis displays the importance scores. Each feature’s importance is represented by a circle, with the size reflecting the score and the exact value labeled within each circle for clarity.

**Figure 6:**
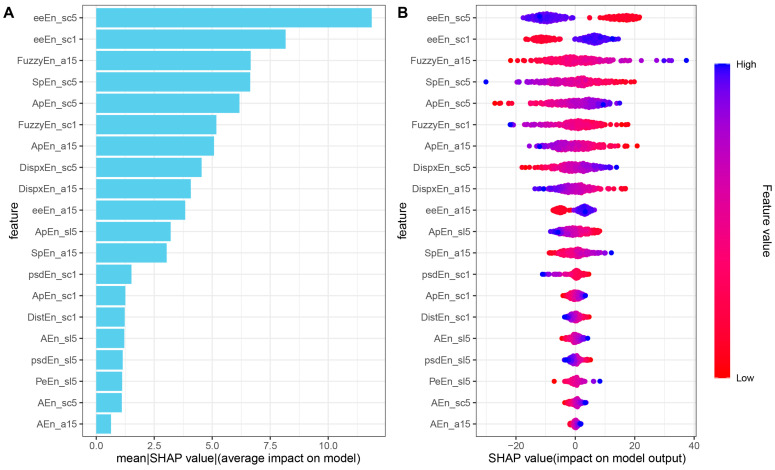
SHAP analysis. (**A**) The importance ranking of the top 20 features according to the mean (|SHAP value|); (**B**) the importance ranking of the top 20 features using the optimal regression model. The suffixes of different features represent various CMSE parameters: “sc1” denotes Scale factor 1, “sc5” denotes Scale factor 5, “a15” represents Area factor 1–5, and “s15” stands for Slope factor 1–5.

We used the optimal regression model (LR), which employed the optimal number of variables (23) obtained from the analysis in Section 3.1, corresponding to the period of 20:00–21:00, to evaluate feature importance. The feature importance calculation is detailed in Section 2.5. The findings, as depicted in Figure 5, show the relative significance of each feature in enhancing model performance (note: area 6–20 was not successfully iterated). Among the CMSE features, scales 1 and 5 demonstrate the most potent predictive capabilities for LVEF levels in hypertensive patients. Additionally, the inclusion of slopes 1–5 and areas 1–5 substantially enhances the predictive capacity of HRV features for LVEF levels in patients with hypertension. Similarly, the SHAP analysis revealed consistent results (see Figure 6).

## 4. Discussion

This study is among the first efforts to explore the relationship between CMSE-based HRV features and LVEF levels in hypertensive patients using 24 h Holter ECG data. Three key findings emerged: Firstly, ML frameworks with ECG signal characteristics can capture subtle cardiac functional fluctuations, enabling high precision LVEF estimation. Secondly, the optimal timing (20:00 and 21:00) of accurate LVEF estimates based on ECG data may relate to HRV’s circadian rhythm. Thirdly, CMSE-derived features can effectively enhance our machine learning framework’s estimation performance.

First and foremost, this study provides novel evidence for the accurate quantitative estimation of cardiac function changes using ECG, an area where the literature is limited and inconclusive. Few works have used ECG to estimate LVEF as a continuous percentage value. Clinical guidelines often categorize patients based on LVEF using fixed classification boundaries [43], which may overlook slight LVEF differences across patients, leading to imprecise treatment decisions. Regression approaches can reduce the risk of such misclassification [44]. Table 5 lists the regression results of the proposed method compared to several previous studies [15,45,46]. This study outperforms others in estimating LVEF levels of patients. And under similar research conditions, the proposed method demonstrates comparable predictive validity to studies identifying LVEF levels through deep learning methods, demonstrating the advancement of the proposed approach. Specifically, this study utilized a LR model to achieve the best performance within the 20:00 to 21:00 period, with a RMSE of 4.61%. Moreover, the MAE was very low at 3.74%, highlighting its superiority. Additionally, Bland–Altman analysis showed a mean bias of 0.22% and 95% limits of agreement at ±10.61%, indicating a close correspondence between the model’s predictions and actual values. Linear regression, a classic machine learning method, is widely used in clinical practice. In this study, RFE was used to reduce the dimensionality of raw variables. It extracted 23 key features from ECG signals, simplifying the model while retaining core information. Compared to complex models, linear regression, with its simple mathematical form and interpretability, showed stronger generalization ability on the dimensionality-reduced data [47]. It effectively avoided overfitting and achieved excellent regression performance.

Our data indicate that the most accurate estimation of LVEF levels based on HRV features occurs in the early evening (20:00–21:00), late night (03:00–04:00), and early morning hour (05:00–06:00), rather than being uniformly distributed across the 24 h period. Potential reasons for the large errors in previous related studies may include failing to explicitly account for the dynamic changes in HRV throughout the day. HRV reflects the beat-to-beat variation in RR intervals (time between R waves), driven by the complex nonlinear interplay of sympathetic and parasympathetic nerves to maintain homeostasis [48].

Heart rate follows circadian rhythms, peaking in the day and dropping at night, with the lowest rates during sleep and the highest when awake [49]. This pattern reflects the dynamic balance of sympathetic and parasympathetic activities. During sleep, sympathetic activity declines and parasympathetic activity rises, lowering heart rate and increasing HRV complexity. The opposite happens during wakefulness [50]. Therefore, during late night (03:00–04:00), changes in heart function can be effectively captured as HRV complexity remains at its peak.

Heart rate drops significantly in the morning after waking. The “morning shift” of cardiac sympathetic–vagus balance may be related to the occurrence of cardiovascular disease at this time [51]. It creates a distinct local change point marking an instant sympathetic activation and a sharp complexity drop. The opposite happens during sleepiness [50]. During the early morning (05:00–06:00) sleep-to-wake transition, HRV decreases with dropping complexity. In the evening (20:00–21:00) wake-to-sleep transition, HRV increases with rising complexity. Under these great changes in HRV complexity, these intervals may be more closely correlated with the original changes in heart function.

Most cardiovascular events show a bimodal pattern, peaking in the morning (06:00–noon) and evening (18:00–midnight), indicating higher cardiovascular risk during these periods, closely linked to changes in HRV complexity [52]. By capturing HRV’s dynamic changes across different times, CMSE analysis offers a more precise cardiac function assessment and provides a new angle for CVD prevention and management.

In addition to identifying effective estimation time intervals, this study also discovered the importance of CMSE-based on HRV data in estimating LVEF levels. We analyzed the feature importance in Section 3.4, which represents the wake–sleep transition period (20:00 to 21:00). The results suggest that CMSE captures LVEF changes in hypertensive patients from more angles compared to single-scale entropy. Among all CMSE features, scales 1 and 5 have the best predictive power for LVEF levels in hypertensive patients, and Scale 5 performed somewhat better. It is reasonable that the complexity of HRV at longer timescales may more accurately reflect certain behaviors of systemic hemodynamic turbulences [53]. Furthermore, slopes 1–5 and areas 1–5 effectively enhance predictive ability of HRV features for LVEF levels in hypertensive patients. During the wake–sleep transition, the gradual decline in sympathetic activity alongside the increase in parasympathetic activity triggers notable short-term fluctuations in cardiac rhythm complexity [50]. Areas 1–5 capture these fluctuations by integrating information from multiple short-term CI. Simultaneously, rapid shifts in autonomic nervous activity manifest as steep trends in heart rate complexity, enabling slopes 1–5 to sensitively detect such changes. Therefore, as is consistent with previous studies, areas under the curve or changes in slope at specific sequence scales can provide better capabilities for detecting diseases and predicting prognosis [54,55]. In this study, we demonstrate the advantages of CMSE analysis in cardiac function detection.

## 5. Limitations

While this study provides valuable insights into the relationship between HRV features and LVEF levels in hypertensive patients, there are opportunities for further improvement in future research. First, the current study focused on the correlation between HRV features and LVEF through CMSE analysis. Future work could expand the scope by including additional echocardiographic parameters such as left ventricular mass index, which are also critical for comprehensive cardiac function assessment. This would provide a more comprehensive understanding of how HRV features relate to various aspects of cardiac health. Second, regarding the sample size, while the current cohort was drawn from communities in a large city in South China, the findings offer a solid foundation for further exploration. Ongoing efforts are underway to expand the cohort by recruiting new participants and planning a multi-center study to enhance the generalizability of our machine learning framework.

Additionally, our team is currently conducting longitudinal studies with 1- to 10-year follow-ups to investigate the long-term relationship between HRV and the progression of hypertension. These studies are expected to yield promising insights into the dynamic interplay between HRV and cardiovascular health, further enriching the understanding of HRV’s role in predicting cardiac functional outcomes. Through these continued efforts, we aim to build upon the current findings and contribute to the development of more comprehensive and widely applicable approaches for cardiovascular assessment.

## 6. Conclusions

Our findings demonstrate the promise of CMSE-based HRV features from Holter ECG as a non-invasive, cost-effective, and interpretable solution for LVEF assessment in patients with hypertension. From a machine learning perspective, the proposed method emphasized CMSE’s clinical potential in capturing autonomic dynamics and cardiac function fluctuations, offering the potential for hypertension management and CVD prevention in community scenarios.

## Figures and Tables

**Table 1:** Baseline characteristics of hypertensive patients.

Characteristics	Value
Age (years)	59.4 ± 9.3
Sex (male), *n* (%)	102 (51.0)
Diabetes mellitus, *n* (%)	157 (78.5)
Working type (day work), *n* (%)	169 (84.5)
Hypertension history (years)	7.5 ± 6.8
LVEF (%)	65 ± 7

**Table 2:** Extracted features and calculation methods.

Features	Calculation Methods
Power spectral entropy	$-\sum_{i=1}^{N}p_{i}\log_{2}p_{i}$, where *p_i_* is the normalized power spectral coefficient.
Energy entropy	$-\sum_{i=1}^{N}e_{i}\log_{2}e_{i}$ , where *e_i_* is the normalized energy coefficient.
Approximate entropy	$\frac{\log C_{m}\left(r\right)}{\log C_{m+1}\left(r\right)}$, where *C*_m_(*r*) is the number of m-length sequences within a tolerance *r* of each other.
Sample entropy	$-\frac{\log\left(\frac{B_{m}\left(r\right)}{A_{m}\left(r\right)}\right)}{\log\left(1/\epsilon \right)}$, where *A_m_*(*r*) and *B_m_*(*r*) are the counts of *m*-length and (*m* + 1)-length similar sequences, respectively.
Fuzzy entropy	$\frac{\log\left(\frac{N_{m}\left(r\right)}{N_{m+1}\left(r\right)}\right)}{\log\left(1/\epsilon \right)}$, where *N_m_*(*r*) and *N*_*m*+1_(*r*) are the counts of *m*-length and (*m* + 1)-length fuzzy similar sequences, respectively.
Permutation entropy	$-\sum_{i=1}^{N}p_{i}\log_{2}p_{i}$, where *p_i_* is the probabilities of the permutation pattern.
Attention entropy	$-\sum_{i=1}^{N}a_{i}\log_{2}a_{i}$, where *a_i_* is the attention coefficient.
Bubble entropy	$-\sum_{i=1}^{N}b_{i}\log_{2}b_{i}$, where *b_i_* is the normalized bubble count.
Dispersion entropy	$-\sum_{i=1}^{N}d_{i}\log_{2}d_{i}$, where *d_i_* is the normalized dispersion coefficient.
Distribution entropy	$-\sum_{i=1}^{N}d_{i}\log_{2}d_{i}$, where *d_i_* is the normalized distribution coefficient.
Gridded distribution entropy	$-\sum_{i=1}^{N}g_{i}\log_{2}g_{i}$, where *g_i_* is the normalized gridded distribution coefficient.
Incremental entropy	$-\sum_{i=1}^{N}i_{i}\log_{2}i_{i}$, where *i_i_* is the normalized incremental coefficient.
Phase entropy	$-\sum_{i=1}^{N}\phi_{i}\log_{2}\phi_{i}$, where *ϕ_i_* is the normalized phase coefficient.
Slope entropy	$-\sum_{i=1}^{N}s_{i}\log_{2}s_{i}$, where *s_i_* is the normalized slope coefficient.
Symbolic dynamic entropy	$-\sum_{i=1}^{N}s_{i}\log_{2}s_{i}$, where *s_i_* is the probabilities of the symbolic sequence.

**Table 3:** Average RMSE across 24 h interval, utilizing regression models with best feature subset (%).

Time	GPR	KNN	LR	MLP	RF	SvmLinear	SvmPoly	SvmRadial	Treebag	CNN
1	5.45	5.38	5.21	5.33	5.41	4.95	5.16	5.27	5.36	5.13
2	5.21	5.61	5.25	5.21	5.48	5.08	5.21	5.31	5.61	5.19
3	5.03	5.45	5.26	5.31	5.50	5.35	5.31	5.19	5.45	6.62
**4**	5.15	5.00	**4.64**	5.32	5.38	5.11	5.29	5.11	5.44	5.02
5	5.11	5.21	5.25	5.15	5.18	5.19	5.21	5.09	5.17	5.15
**6**	5.05	5.10	5.17	5.83	5.25	5.01	5.28	5.17	**4.67**	5.25
7	5.25	5.54	5.26	5.22	5.68	5.39	5.27	5.24	5.35	5.32
8	5.64	5.80	5.24	6.43	5.36	5.20	5.30	5.43	5.16	5.31
9	5.10	5.53	5.00	5.50	5.79	4.99	5.22	5.48	5.66	5.13
10	5.38	5.52	5.17	6.07	5.59	5.39	5.28	5.30	5.27	5.29
11	5.66	5.44	5.20	5.35	5.54	4.93	5.30	5.39	5.36	5.31
12	5.59	5.53	5.31	5.36	5.56	5.38	5.29	5.38	5.52	5.30
13	5.54	5.38	5.52	5.11	5.63	5.35	5.29	5.38	5.48	5.30
14	5.34	5.48	5.18	5.01	5.62	5.27	5.23	5.22	5.11	5.15
15	5.06	5.25	5.23	5.34	5.34	5.11	5.24	5.27	4.97	5.25
16	5.52	5.14	5.06	5.53	5.33	5.25	5.18	5.12	5.38	5.07
**17**	5.14	4.97	4.95	5.44	4.97	4.81	**4.80**	4.90	5.13	4.92
18	5.60	5.51	5.21	5.76	5.54	5.34	5.28	5.31	5.34	5.31
19	5.51	5.50	5.17	5.64	5.53	5.31	5.27	5.43	5.13	5.21
20	5.37	5.45	5.21	5.88	5.32	5.04	5.24	4.90	5.27	5.25
**21**	4.64	5.22	**4.61**	5.50	4.85	4.92	5.22	4.84	4.93	4.81
22	5.22	5.20	5.13	5.37	5.36	5.02	5.14	5.10	5.11	5.15
23	5.28	5.15	5.25	5.36	5.29	5.11	5.23	5.24	5.34	5.16
24	5.34	5.38	5.06	5.55	5.54	5.31	5.31	5.23	5.22	5.51

Note: The bold numbers indicate the four time periods with the minimum RMSE values and their corresponding models within the 24 h interval.

**Table 4:** Average MAE across 24 h interval, utilizing regression models with best feature subset (%).

Time	GPR	KNN	LR	MLP	RF	SvmLinear	SvmPoly	SvmRadial	Treebag	CNN
1	4.54	4.42	4.10	4.37	4.51	4.20	4.30	4.34	4.21	4.30
2	4.27	4.39	4.27	4.35	4.46	4.30	4.34	4.34	4.38	4.34
3	4.22	4.41	4.27	4.36	4.36	4.40	4.36	4.13	4.30	5.24
**4**	4.16	3.93	**3.84**	4.35	4.30	4.05	4.34	4.19	4.47	4.12
5	4.27	4.29	4.19	4.25	4.29	4.32	4.33	4.25	4.24	4.27
**6**	4.20	4.19	4.23	4.68	4.25	4.06	4.33	4.26	**3.80**	4.29
7	4.20	4.28	4.25	4.11	4.50	4.22	4.21	4.15	4.29	4.21
8	4.60	4.53	4.25	4.88	4.31	4.26	4.32	4.40	4.11	4.37
9	4.10	4.45	4.01	4.45	4.68	4.13	4.29	4.35	4.38	4.20
10	4.48	4.50	4.23	4.88	4.60	4.41	4.32	4.29	4.42	4.33
11	4.49	4.29	4.20	4.35	4.37	4.17	4.32	4.29	4.18	4.30
12	4.53	4.56	4.35	4.42	4.41	4.34	4.25	4.36	4.48	4.36
13	4.36	4.39	4.72	4.20	4.47	4.26	4.26	4.29	4.48	4.29
14	4.32	4.29	4.18	4.32	4.60	4.28	4.19	4.27	4.28	4.15
15	4.02	4.21	4.21	4.37	4.43	4.10	4.26	4.15	4.07	4.23
16	4.44	4.14	4.11	4.46	4.30	4.06	4.17	4.13	4.24	4.05
**17**	4.23	4.06	3.99	4.29	4.17	**3.92**	3.99	3.99	4.12	4.30
18	4.59	4.42	4.22	4.52	4.57	4.30	4.32	4.32	4.30	4.33
19	4.42	4.30	4.14	4.46	4.42	4.32	4.32	4.39	4.11	4.27
20	4.24	4.44	4.11	4.71	4.39	4.05	4.23	3.83	4.29	4.11
**21**	3.86	4.16	**3.74**	4.45	4.00	4.17	4.25	3.98	3.99	4.06
22	4.21	4.20	4.18	4.57	4.34	3.96	4.21	4.19	4.13	4.20
23	4.05	3.95	4.27	4.39	4.11	4.09	4.27	4.06	4.13	4.10
24	4.25	4.43	3.97	4.47	4.54	4.24	4.36	4.13	4.24	4.50

Note: The bold numbers indicate the four time periods with the minimum MAE values and their corresponding models within the 24 h interval.

**Table 5:** Related work on estimating LVEF by ECG in patients.

Author	ECG Duration	Data	Features	Regression Model	RMSE (%)	Bland–Altman Mean ± 1.96 SD (%)	MAE (%)
Mohanad et al. (2021) [45]	1 h (24 h study)	America, 92 (Heart Failure)	HRV parameters	SVM	10.4	0.53 ± 20.44	NA
Luiz et al. (2021) [46]	15 min	Brazil, 63 (Chagas disease)	HRV parameters	RF/MLP/ KNN/SVM	NA	−0.48 ± 23.53	NA
Akhil et al. (2022) [15]	5 s	America, 219,437 (Normal)	ECG features	CNN	NA	NA	6.14
Current study	1 h (24 h study)	China, 200 (Hypertension)	CMSE-based HRV features	GPR/KNN/LR/ MLP/RF/SVM/Treebag/CNN	4.61	0.22 ± 10.61	3.74

## Data Availability

The data presented in this study are available on request from the corresponding author. The data are not publicly available due to issues of participant confidentiality.

## Supplementary Materials

The following supporting information can be downloaded at: https://www.mdpi.com/article/10.3390/bios15070442/s1.
