# Supplementary material for: Utilizing Circadian Heart Rate Variability Features and Machine Learning for Estimating Left Ventricular Ejection Fraction Levels in Hypertensive Patients: A Composite Multiscale Entropy Analysis
Source: Biosensors (Basel). 2025 Jul 10;15(7):442. doi: 10.3390/bios15070442 (PMC12293304; doi:10.3390/bios15070442)
Supplement: Supplementary file 1 [file biosensors-15-00442-s001.zip › biosensors-3609485-supplementary.pdf]

The scale factors were further categorized into five different parameters:

1. The value of entropy at scale 1. Its calculation formula is as follows:

$$E(1) = \text{Entropy}\left(y_{1,j}^{(1)}\right)$$

2. The value of entropy at scale 5. Its calculation formula is as follows:

$$E(5) = \frac{1}{5}\sum_{k=1}^{5} \text{Entropy}\left(y_{k,j}^{(5)}\right)$$

3. Slope 1–5: The linear-fitted slope between scales 1 and 5. Its calculation formula is as follows:

$$Slope1 - 5 = \frac{E(5) - E(1)}{5 - 1}$$

4. The area under the curve between scales 1 and 5 (Area 1–5), which serves as a measure of complexity across short timescales, also known as the short-term Complexity Index (CI). Its calculation formula is as follows:

$$Area1 - 5 = \sum_{\tau=1}^{5} E(\tau)$$

5. The area under the curve between scales 6 and 20 (Area 6–20), which serves as a measure of complexity across long timescales, also known as the long-term CI. Its calculation formula is as follows:

$$Area6 - 20 = \sum_{\tau=6}^{20} E(\tau)$$
